# Substance use during COVID-19 pandemic: impact on the underserved communities

**DOI:** 10.15190/d.2021.20

**Published:** 2021-12-31

**Authors:** Natalia C. Chacon, Namrata Walia, Abigail Allen, Anthony Sciancalepore, Joyce Tiong, Rachel Quick, Sanjana Mada, Miguel A. Diaz, Ivan Rodriguez

**Affiliations:** ^1^The University of Texas Health Science Center School of Public Health, Houston, TX, USA; ^2^Family and Community Medicine, Baylor College of Medicine, Houston, TX, USA; ^3^University of Texas at San Antonio, TX, USA; ^4^Paul L Foster School of Medicine, El Paso, TX, USA; ^5^Department of Family Medicine, Larkin Community Hospital, Florida, USA

**Keywords:** Substance use, SARS-CoV-2, pandemic, disparities.

## Abstract

The number of overdose deaths are on the rise all over the world. An estimate of 93,000 drug overdose deaths have been estimated in the United States in 2020. COVID-19 pandemic has exacerbated the drug crisis. Factors, such as existing health disparities among underserved communities, lack of resources for people of color, lack of belief in available resources, social isolation and economic burden, limited access to treatment, regulatory barriers in telehealth, and stress from the on-going COVID-19 pandemic have been identified as some of the key factors behind the acute health effects of people with substance use disorder. These interrelated factors exacerbate the impact of already existing disparities in the underserved communities. Policy and regulatory changes around telehealth and access of treatment for substance use disorder are warranted. Evidence-based strategies and other safer drug practices should be implemented to mitigate the impact on human health. Investment in programs that increase access to treatment, will be useful for potential future pandemics, where increasing mental health services and overall access to healthcare in disadvantaged communities would lessen the disparities in physical and mental ailments. In this review, we are evaluating and summarizing the acute health effects of the ongoing COVID-19 pandemic on individuals with substance use disorder.

## 1. Introduction

Since the onset of the COVID-19 pandemic, there have been various impacts on societal well-being; not only have many people had to face the potentiality of COVID-19 infection and death, but they have also had to struggle through economic collapse and social isolation due to quarantine measures. In correlation with these problems, there has been a significant increase in those reporting symptoms of mental illness. Compared to 1 in 10 adults in 2019, 4 in 10 American adults have reported symptoms of anxiety or depression throughout the course of the pandemic^1^. Given that up to 50% of those with substance use disorders (SUDs) experience symptoms of mental illness, reports of substance abuse have been on the rise as well. Substance use disorder, also known as drug addiction, occurs when a physiological or psychological dependence on a drug develops that leads to an inability to control one’s use. According to the Centers for Disease Control and Prevention (CDC), 13% of Americans have reported increasing or starting substance use as a way of coping with stress related to the pandemic^2^. An overdose reporting system known as ODMAP also reported an 18% increase nationwide in opiate overdoses^2^. As noted by behavioral psychologist William Stoops, “There’s sort of a perfect storm of factors that we know increase drug use. People are stressed and isolated, so they make unhealthy decisions, including drinking and taking drugs”^2^. Statistics that show significant increases after COVID-19’s initial outbreak demonstrate that the pandemic is likely having an influence on mental illness and substance abuse that warrants further investigation. Closer analysis of this influence reveals a disproportionate impact on these indicators in disadvantaged communities with preexisting health inequities. Throughout the pandemic, African American and Hispanic populations have been found to be three times more likely to be hospitalized by COVID-19^3^ . This is likely the result of a myriad of factors, including lack of access to healthcare/mental health services and exposure to poor socioeconomic conditions made even worse by the pandemic. When combined with the preexisting inequities and systemic issues that these communities face, mental health and substance abuse impacts are sure to be exacerbated. This paper attempts to explore this interplay of factors and provide policy recommendations geared towards mitigating the pandemic's impact on substance abuse, mental health, and exacerbation of health inequities. In order to explore the influence of the pandemic on exacerbating substance abuse in disadvantaged communities, this paper inspects the problem through the lens of acute human health effects. Considering that the pandemic has only been ongoing for a limited period of time, studying the acute effects of the pandemic is likely the best way to represent its burden on substance abuse since COVID-19’s initial outbreak. To accurately differentiate the issues contributing to the disproportionalities in underserved communities, the acute health effects from before and after the pandemic will be examined. This will provide a clearer picture as to how the pandemic influenced mental health and substance abuse in 2020, while also highlighting the inequities that underserved communities face. As one can imagine, the issue at hand is multifactorial and has a litany of influencers. The causal loop diagram listed below provides an illustration of how these factors may work together and how one can intervene via policy implementation to mitigate outside factors on substance abuse. Despite the ongoing pandemic, simply identifying problems from a system’s thinking approach can go a long way in reducing the burden of mental illness and substance abuse in disadvantaged communities and in society at-large.

## 2. Substance Use during the COVID-19 pandemic and its effects on the underserved communities

The burden of Substance Use Disorder (SUD) has been increasing in the United States for decades among both adults and adolescents^4^. In 2018, around 20.3 million people living in the United States 12 years or older reported having an SUD^5^. Of these, 14.8 million were related to alcohol abuse and around 8.1 million were related to illicit drug use^5^. Of the 8.1 million with an illicit drug SUD, around 2 million people had an opioid use disorder, second in its category to marijuana use. 63.6% of people with an opioid SUD reported misusing opioids to relieve physical pain. A less common answers included to ‘feel good’ or ‘get high’, to relax, to help with sleep, to experiment, to help with feeling emotions, and because they were ‘hooked’ or needed to, respectively (Substance Use and Mental Health Services Administration). Other causes of SUDs include environmental factors such as domestic abuse, parental or peer drug use, community attitudes towards drug use, and mental health disorders. Indigenous Americans and Alaskan Natives aged 12 and older had the highest rate of substance abuse in 2018 at 12.8%, followed by white people at 7.7%, 6.8% of African Americans, and 6.6% of Hispanic people^6^. The COVID-19 pandemic has had a dramatic effect on the prevalence of SUDs and treatment options for persons seeking healthcare for acute health complications. Since the onset of COVID-19, there has been a 23% increase in alcohol abuse and a 16% increase in drug abuse for people who had consumed those substances before the pandemic^7^. People in self-isolation reported a 26% higher consumption than they would normally use to cope^7^. In addition to higher consumption among long-time substance users, isolation and COVID-19 related fears have both been associated with limited access to detoxification centers and psychological distress, factors that play a major role in the increase of drug and alcohol use^8,9^. Not only are people at a higher risk of developing a SUD because of the pandemic, but SUDs also put people at a higher risk for COVID-19^10,11^. People with SUDs may currently have or be at a greater risk for respiratory infections, cardiovascular complications, chronic obstructive pulmonary disease (COPD), and other viruses and infections related to weakened immune systems. Opioids act as immune suppressants that can impair the function of macrophages, NK cells, and T-cells^10^. This makes it more difficult for the body to fight the COVID-19 infection when the body’s natural immune defenses are exacerbated. In addition, activation or depression of opioid receptors in the brain can impact respiratory function and the inflammation response, both of which can cause COVID-19 related complications^10^. Individuals with SUDs that have experienced non-fatal overdose are more likely to have chronic conditions that are associated with COVID-19 severity including CPD, diabetes, and heart disease with the adjusted odds ratios of 2.01, 1.24, and 2.08^11^. People with SUDs are at a higher risk for having comorbidities that are associated with COVID-19 and severity of COVID-19, which puts them at increased risk for other acute complications, the most severe of which being death. Overdose rates have largely increased during the COVID-19 pandemic; however, the increase has not been uniform. Opioid overdose rates have increased among African Americans, but they have decreased overall for white individuals. One theory for this sharp contradiction, especially given that white people reported higher usage of opioids than any other race previously, is that the pandemic had worsened determinants such as disproportionate economic deprivation in predominately African American communities and pre-existing racial disparities in accessing treatment care^12^. These claims are not unfounded; historically, in times of crisis the most socially vulnerable populations are more likely to be exposed disproportionately to stressors^13^. African Americans and Hispanic persons were more likely to start or increase substance abuse to cope with increased rates of mental health issues related to COVID-19 than their white counterparts with a prevalence ratio of 1.75 and 2.09 respectively^14^. Preventing overdose in the era of the pandemic has been more difficult than ever and has become even harder to do for communities of color, people of low socio-economic status, and persons with disabilities. Minority populations already experience discrimination, racism, and stigma when seeking healthcare. However, there is a higher chance of those occurrences when race intersects with the stigmas held about people with SUDs^14^. Many treatment facilities had to reduce their hours and services for people with SUDs, increasing the barriers to treatment and placing these individuals at a higher risk for death^15^. Many proposed solutions to the existing gap for treatment involve the use of online or telephone delivery methods to overcome these obstacles^15-17^. Further recommendations are needed to understand how these principles can be applied to close treatment gaps.

## 3. Causal Loop Diagram of the interrelated factors that influence substance abuse

**Figure 1:**
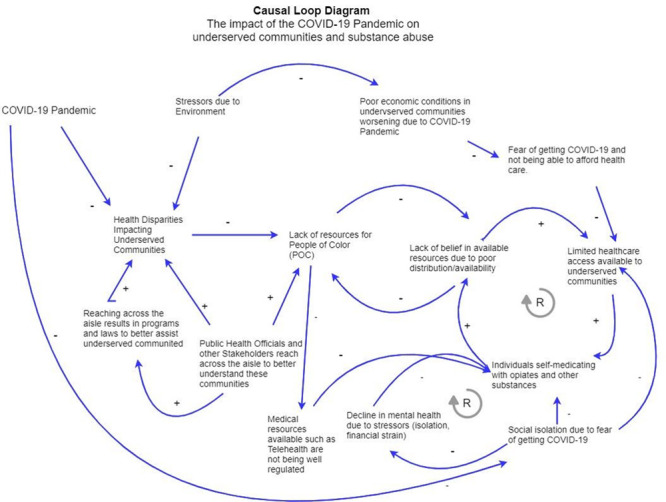
Causal Loop Diagram

Figure 1 shows the comprehensive list of factors that are interrelated. The COVID-19 pandemic has had many implications worldwide, which disproportionately affected some populations more than others based on certain deteminant factors. Not only was there a sense of fear and uncertainty of the infection that spread, but also the secondary effects of the pandemic such as social isolation and economic burdens. These burdens exacerbated the many barriers that have been plaguing society for years, such as limited access to health care, a larger gap on the socioeconomic hierarchy, and increasing rates of anxiety and depression due to lack of social support.

Figure 1 shows the comprehensive list of factors that are interrelated. The COVID-19 pandemic has had many implications worldwide, which disproportionately affected some populations more than others based on certain determinant factors. Not only was there a sense of fear and uncertainty of the infection that spread, but also the secondary effects of the pandemic such as social isolation and economic burdens. These burdens exacerbated the many barriers that have been plaguing society for years, such as limited access to health care, a larger gap on the socioeconomic hierarchy, and increasing rates of anxiety and depression due to lack of social support.

The pandemic, and the accompanying economic stress and need to social distance, negatively impacted mental health, especially among those with SUDs. Nearly 50% of individuals that have SUDs are affected by mental illness, and roughly 13% started using substances again or increased previous usage as a coping mechanism during the pandemic^2^. Moreover, traumatic stress syndromes stemming from the pandemic were found to be significantly associated with substance abuse^7^. The psychological distress during the pandemic along with the existing mental health disorders in individuals with substance abuse disorders has caused a detrimental synergistic effect that increases rates of addiction and risks of overdose. This highlights the need to focus on bettering mental health services and support during adverse public health events, like the COVID-19 pandemic.

The illicit drug that is abused most in those with SUDs are opiates with almost 2 million individuals affected^5^. Many of the individuals who experience SUDs have self-reported their misuse stemming from initial alleviation of physical pain. This is because opioids are the most commonly prescribed pain medication to treat moderate to severe pain with an extremely high abuse potential. It is important to understand that people of color, such as Hispanics and African Americans, have been disproportionately affected by the pandemic, with almost three times more hospitalizations. These minority groups were also reported to be more likely to start or increase abusing substances as a coping mechanism during the pandemic. Racial inequalities like these become more pronounced in a state of environmental disaster due to their limited resources to health care, rehabilitation centers, transportation, and even language barriers, further emphasizing the need to address these disparities in disadvantaged communities.

Another important issue to consider when discussing the acute effects of COVID-19 on SUDs is the opposite relationship. This means exploring the increased susceptibility of COVID-19 in patients that are suffering from SUDs. It has been found that individuals with SUDs are more likely to develop respiratory complications and cardiovascular disease^10^. COVID-19 is especially higher risk in patients that use opiates which impairs the cells of the innate and adaptive immune system leading to more hospitalizations in this subgroup. These increased hospitalizations lead to an overall decrease in the health status especially in communities that are already disproportionately affected by both COVID-19 and SUDs. Thus, the link between COVID-19 and SUDs and their impact on disadvantaged communities demonstrates the importance of addressing health disparities to combat both new and existing public health issues.

Since COVID-19 is a virus that is spread by respiratory droplets or aerosols, it is important to take the necessary precautions to minimize transmission especially in close quarters. Group sessions at many rehabilitation centers involve being seated closely in a small room which does not meet the CDC recommendation to socially distance six feet apart^18^. This has led to the temporary shutdown of many therapy centers. Telemedicine has been implemented in many healthcare settings and has even been a resource for those with SUDs so that counseling and access to a healthcare professional is available to individuals from home^19^. While this has had promising approaches, it is important to communicate to patients from all communities of their options to minimize the rates of overdose deaths due to lack of resources.

Since the biggest factor of increasing rates of SUDs has been over prescription of controlled substances, it is better to mitigate the problem from beginning upstream which is with physicians. This includes setting more stringent policies in place in the prescription of opioids and other highly addictive medications and monitoring patients closely to prevent tolerance buildup. Other upstream efforts include medical education and increasing health literacy in order to de-stigmatize societal expectations around drug abusers. By creating interventions that educate communities about the dangers of drug abuse and proper use, it is possible to lower the rates of SUDs before they progress to a disorder^20^. These upstream efforts can alleviate the detrimental effects of COVID-19 by increasing the health status of those suffering from SUDs and provide the medical education necessary to find safer coping mechanisms during a time when psychological distress is high.

## 4. Recommendations

As discussed above, there has been an exponential increase in substance use and related overdose deaths during pandemic. In the beginning of the pandemic, many outpatient clinics decreased their in-person appointments leading to widening of the existing treatment implementation gap. This is the gap of those who need treatment for a substance use disorder and few who receive it. Telehealth reduced disparity in the access of treatment by increasing availability and reach of treatment. New regulations and guidelines, including Medicare coverage of telehealth services^18^, were implemented to remove barriers in providing healthcare services using telehealth^21^. The delivery of healthcare using technology proved to be an efficient tool to expand services to rural areas^19^. However, use of telemedicine was restricted to patients with mild cases of SUD^10^ leading to higher hospitalizations and/or overdose deaths. Although the use of telemedicine did not help with equitable healthcare services, it is a promising seamless access for many medical needs, especially during crisis like pandemic. In the future, policymakers and insurance regulators should work towards designing an optimal payment policy for telehealth services to increase access to telemedicine post pandemic.

The underserved communities have been disproportionately affected by the acute effects of substance use during the on-going pandemic. The disparity in the health outcomes among the various races has been attributed to inequitable distribution of healthcare resources. Studies conducted before the pandemic have reported federal and economic policies and regulations, environmental context and community level operation as some of the reasons behind less specialty care for Blacks and Hispanics compared to White population^22^. Changes in regulation such as lifting the X-waiver requirement resulted in an increase of buprenorphine access by increasing the pool of buprenorphine prescribers^23^. X waiver is a waiver to Drug Addiction Treatment Act of 2000 that authorizes use of buprenorphine. The requirement was lifted during the COVID-19 pandemic. This is indicative of the need to increase the access of treatment for patients with SUD. Removing the waiver will lead to equitable and accessible treatment for patients in low-income households and in rural areas^24^. Many healthcare providers and policymakers are promoting “X the X-waiver” movement to promote deregulation of this requirement leading to a long-term better access and destigmatized treatment^25^.

Physicians have recommended a full range of treatment options for newly diagnosed patients with substance use to ensure better medication adherence. Additionally, it has been recommended to frequently dispense methadone and decrease urine toxicology screening^20^. Although some studies have reported provider hesitancy citing it as risky^26^. But, if deemed safe, this could be a step towards increased availability of treatment and improved health equity. Moreover, it has been recommended to increase methadone availability through either home delivery or community pharmacies.

The socio-economic factors responsible for health disparity in substance use are multi-faceted. Public health and harm reduction programs can help in reducing and de-stigmatizing community and societal factors. These programs teach the community about safer drug use. Poly-drug use (mixing of more than 1 kind of substance) can have hazardous drug effects, sometimes leading to death. Through these outreach programs, people are educated on lethal effects of mixing certain drugs. During the pandemic, such practices emerged online like virtual peer supervision while using injections^20^. Similarly, syringe exchange programs distribute sterile syringes to drug users for safer drug practices^27^. It has been legalized in some states and promotes preventive measures.

To truly bring forth the change, all the key stakeholders must be properly engaged. The groups such as political leaders, medical professionals, nonprofits that focus on substance use, and public health agencies need to reach across the aisle to better understand why disadvantaged communities are turning to substance use as a way to cope with the pandemic. Not properly engaging disadvantaged communities would further perpetuate the current systems that have hindered these communities from getting the assistance they deserve and need. One major stakeholder in this process is the healthcare system and its individual employees. COVID-19 closures and overall lack of access to healthcare in disadvantaged communities have been a major contributor to poorer outcomes regarding COVID-19 and an overreliance on self-medication. These communities may also have a harder time accessing mental health services, which may create conditions that are more likely to lead to substance abuse. This is where other relevant stakeholders, like the Substance Abuse and Mental Health Services Administration (SAMHSA), could play a vital role. Investments in programs that increase access to treatment will be useful during potential future pandemics, where programs increasing mental health services and overall access to healthcare in disadvantaged communities would lessen the disparities regarding physical and mental ailments.

Summarizing the recommendations made:

- Increase access to telemedicine post pandemic

- Continue waiving the X-waiver requirement post pandemic to improve access to care

- Frequently dispense methadone and decrease urine toxicology screening

- Increase methadone availability through either home delivery or community pharmacies

- Introduce harm reduction programs in the communities

- Increase funding for community outreach programs

## 5. Conclusion

The COVID-19 pandemic significantly challenged our society, consequently resulting in increased mental health struggles along with increased substance abuse. There are many different factors that are attributed to these issues, most notably, economic collapse, social isolation, and lack of access to healthcare. These factors concentrated on lower income communities that have continuously suffered from pre-existing inequities that make it much harder to avoid the effects of increased substance abuse in the community and the areas surrounding. Substance Use Disorders (SUD’s) have been increasing in the United States for decades, yet few advances have been made in preventing the spread. Mental health and stress have a direct correlation with the prevalence of substance abuse, due to the continued lack of resources and support throughout the pandemic, these rates were difficult to control. Due to the sheer isolation from the pandemic, there was an increase, or reintroduction in a lot of cases, of mental illnesses; mental illness is one of the largest contributors to people either beginning to use substances or returning to substance use. One major factor, as discussed in the literature review, was the treatment gap. To combat the treatment gap there was an overwhelming shift to telemedicine during this time, but telemedicine unfortunately does not and could not reach these underserved communities who lacked things such as health insurance or the technology to receive this care. As more resources and relief came out during the pandemic to combat these consequential stressors, few actually benefited communities where it was most needed. In times of crisis, we continue to see these vulnerable populations be disproportionately affected by these stressors. To prevent this increase of overdose and substance abuse many different groups must come together (ie. local and state governments, health care workers, law enforcement) in order to further prevent the consequences from stressors resulting from COVID-19. The acute health effects resulting from the pandemic give us a clear answer on how we should align our efforts politically and socially to prevent further damage. Being prepared as a whole for adverse events, allow us to also be prepared for adverse outcomes. This issue is multifactorial and cannot be pinpointed on one singular stressor, but by planning in a way that diminishes the effects of a future pandemic, we can avoid the consequences such as these. Recommendations from this study conclude more specifically on areas of improvement needed to combat substance abuse following adverse events.

## KEY POINTS

*◊ There has been an exponential increase in substance use and related overdose deaths during COVID-19 pandemic.*

*◊ Telehealth is bridging the gap & reaching out to people in rural areas seeking treatment*

*◊ Changes in healthcare policies related to the substance use have increased the access to treatment*

## OPEN QUESTIONS

*◊ Will the waiving of X waiver program stay beyond pandemic?*

*◊ Are there any other strategies to combat substance abuse following adverse events?*
